# Mosaicism in Short Tandem Repeat Disorders: A Clinical Perspective

**DOI:** 10.3390/genes16020216

**Published:** 2025-02-13

**Authors:** Rose M. Doss, Susana Lopez-Ignacio, Anna Dischler, Laurel Hiatt, Harriet Dashnow, Martin W. Breuss, Caroline M. Dias

**Affiliations:** 1 Section of Genetics and Metabolism, Department of Pediatrics, University of Colorado Anschutz Medical Campus, Aurora, CO 80045, USA; 2 Department of Human Genetics, University of Utah School of Medicine, Salt Lake City, UT 84132, USA; 3 Department of Biomedical Informatics, University of Colorado Anschutz Medical Campus, Aurora, CO 80045, USA; 4 Section of Developmental Pediatrics, Department of Pediatrics, University of Colorado Anschutz Medical Campus, Aurora, CO 80045, USA

**Keywords:** genomic mosaicism, methylation mosaicism, short tandem repeats, *FMR1*, *HTT*, *DMPK*, fragile X, Huntington disease, myotonic dystrophy type 1

## Abstract

Fragile X, Huntington disease, and myotonic dystrophy type 1 are prototypical examples of human disorders caused by short tandem repeat variation, repetitive nucleotide stretches that are highly mutable both in the germline and somatic tissue. As short tandem repeats are unstable, they can expand, contract, and acquire and lose epigenetic marks in somatic tissue. This means within an individual, the genotype and epigenetic state at these loci can vary considerably from cell to cell. This somatic mosaicism may play a key role in clinical pathogenesis, and yet, our understanding of mosaicism in driving clinical phenotypes in short tandem repeat disorders is only just emerging. This review focuses on these three relatively well-studied examples where, given the advent of new technologies and bioinformatic approaches, a critical role for mosaicism is coming into focus both with respect to cellular physiology and clinical phenotypes.

## 1. Introduction

Short tandem repeats (STRs), also referred to as microsatellites, are 1–6 base pair regions of repetitive DNA, accounting for approximately 3–5% of the human genome [1]. Tandem repeat disorders (TRDs), or repeat expansion disorders (REDs), occur when a tandem repeat expands or contracts from a benign to a pathogenic range, resulting in loss-of-function or toxic gain-of-function effects [2,3]. There are more than 60 known human TRDs, including fragile X syndrome (FXS), Huntington disease (HD), spinocerebellar ataxia, and myotonic dystrophy type 1 (DM1) [4,5,6]. Additionally, tandem repeat expansions have been linked to autism, Parkinson’s disease, schizophrenia, and cancer [7,8,9,10,11,12]. Recently, genome-wide analyses have identified a role for STRs in gene expression, higher-order chromatin organization, and complex traits such as height and biomarkers of health [13,14,15,16,17]. Thus, STRs play central and critical roles in the cellular physiology of human health and disease.

Compared to most of the human genome, STRs exhibit higher mutation rates and instability [18,19]. In addition to germline instability, STRs also exhibit instability in somatic tissue, particularly when they exceed a pathogenic threshold: STRs can expand, contract, and acquire sequence changes. In some cases, this somatic repeat number instability may drive disease progression. Somatic alterations in epigenetic marks in different cells of the body within the same individual can also occur. For example, DNA methylation is a critical epigenetic signature at GC-rich CpG islands that can inactivate gene expression [20]. Length and methylation mosaicism lead to significant cellular variability and have important consequences for cellular pathophysiology and clinical phenotypes. However, given historical technical limits, our understanding of the role of mosaicism in STR-linked disorders is only just emerging.

Over one million variable STR loci have been identified in the human genome, most of which are found in noncoding regions, with only 8% in coding regions [21,22]. However, coding or gene proximal STR loci contribute disproportionately to human disease. The objective of this review is to explore the pathogenic impact of STR mosaicism with a specific focus on *FMR1*, *HTT*, and *DMPK*, i.e., fragile X, HD, and DM1. These are clinically well-described TRDs, and there is an emerging appreciation for the role of mosaicism in these disorders from both the cellular and clinical perspectives.

## 2. Tandem Repeat Disorders

**Figure 1:**
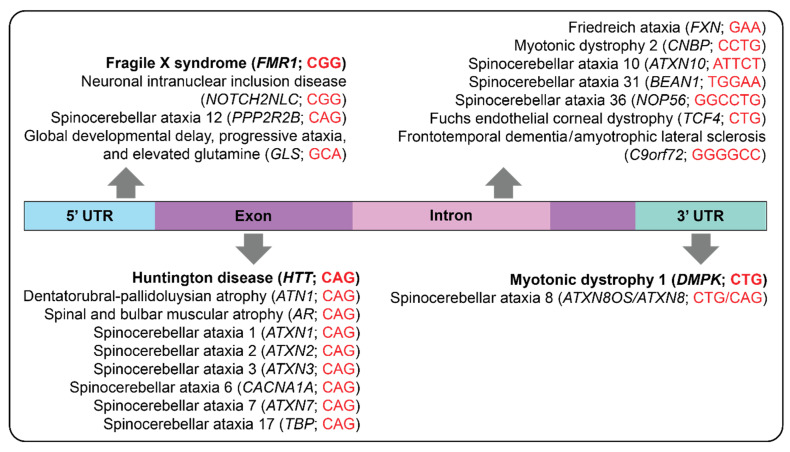
Genetic location and short tandem repeat motif associated with example disorders. The majority of known STR disorders are found within the exonic and intronic regions of the affected gene. Disorders of interest for this review are distinguished in bold. *FMR1*; #300624, *NOTCH2NLC*; #603472, *PPP2R2B*; #604326, *GLS*; #618412, *HTT*; #143100, *ATN1*; #125370, *AR*; #313200, *ATXN1*; #164400, *ATXN2*; #183090, *ATXN3*; #109150, *CACNA1A*; #183086, *ATXN7*; #164500, *TBP*; #607136, *FXN*; #229300, *CNBP*; #602668, *ATXN10*; #603516, *BEAN1*; #117210, *NOP56*; #614153, *TCF4*; #613267, *C9orf72*; #105550, *DMPK*; #160900, *ATXN8OS/ATXN8*; #608768. # = Phenotype MIM number..

As mentioned above, there are more than 60 known TRDs in humans. While they can greatly differ in the length and composition of their repeat unit, common themes emerge (Figure 1). For instance, some neurodegenerative disorders are driven by polyglutamine tracks [23]. The overall landscape of TRDs has been expertly reviewed before [24]. Here, we focus on a summary of fragile X, HD, and DM1 as they are most pertinent to our discussion of STR mosaicism. However, there is growing appreciation for somatic instability in additional TRDs [25,26]. Additionally, distinct repetitive DNA elements, such as variable number tandem repeats, have also been linked to neurological disease, but this is beyond the scope of this review.

### 2.1. Fragile X-Related Conditions

Fragile X-related conditions are caused by CGG expansions positioned in the 5′ untranslated region (UTR) of *FMR1* (fragile X messenger ribonucleoprotein 1), located on the X chromosome [27,28,29,30,31,32]. Among the general population of unaffected individuals, the CGG motif ranges between approximately 5 and 40 repeats. Intermediate alleles, or gray zone alleles, of approximately 41–54 repeats, are not classically considered to confer risk for fragile X-related conditions the way the full mutation and premutation do. Studies seeking to confirm separate associations of these gray zone alleles with distinct clinical outcomes, such as Parkinsonian symptoms and higher risk of death, have met with conflicting results [27,33]. Thus, the clinical impact of gray zone alleles remains controversial. The premutation range, which is ~55–200 repeats, is characterized by increased *FMR1* mRNA [28] but variably reduced *FMR1* protein (FMRP). The premutation is associated with an increased risk of acquiring multiple adult-onset conditions, including fragile X-associated primary ovarian insufficiency (FXPOI), fragile X-associated tremor/ataxia syndrome (FXTAS), and fragile X-associated neuropsychiatric disorders (FXAND) [28,29]. There are a variety of hypotheses surrounding the cellular pathophysiology of FXTAS, which are described extensively elsewhere, including DNA damage and aberrant gain of function toxic RNA and peptide formation [30,31]. Moreover, the premutation allele is particularly unstable in the human female germline, resulting in increased risk in offspring with the full mutation [32], described below.

The *FMR1* full mutation is more than 200 CGG repeats in length, which leads to the well-characterized neurodevelopmental disorder FXS [34]. FXS results from hypermethylation of the CGG repeats, leading to the inactivation of *FMR1* and absent FMRP, a protein crucial during brain development [35,36]. This disorder is a leading inherited form of intellectual disability and has been described as the most common single-gene cause of autism, accounting for 1–6% of autism cases [37]. Other notable symptoms include hyperactivity, impulsivity, anxiety, poor language development, and epilepsy [37]. FXS is inherited in an X-linked dominant pattern. Both males and females can be affected, albeit with differences in severity and progression, much of which is attributed to stochastic patterns of X-chromosome inactivation in females [38,39]. Interestingly, although there are also neurodevelopmental phenotypes, in general, the premutation does not cause FXS per se, and the full mutation does not lead to premutation-associated disorders, implicating unique cellular pathophysiology in each condition.

### 2.2. Huntington Disease

HD is an autosomal dominant progressive neurodegenerative disorder caused by a CAG repeat expansion in the first exon of *HTT*, located on chromosome four [40]. This coding repeat expansion produces a polyglutamine (polyQ) tract in the protein product. Characteristics of HD include uncontrollable motor movements, psychiatric symptoms, and progressive cognitive decline [41]. *HTT* encodes huntingtin, a widely expressed brain protein, which plays a role in brain development, vesicle and organelle trafficking, and neuroprotection [42,43,44,45,46]. In HD, the CAG repeat-associated polyglutamine tract leads to the formation of insoluble aggregates, which have been found in the striatum, cerebellum, spinal cord, and cortex [47]. These aggregates have also been found in neuronal processes, potentially disrupting synaptic transmission [48]. Striatal projection neurons are uniquely vulnerable, with specific degeneration of these cell types observed in HD neuropathology [49].

At the HD locus, unaffected individuals have 10–35 CAG repeats. Those with complete penetrance of the disease have more than 40 repeats, while incomplete penetrance is associated with an expansion size ranging from 36 to 39 [50]. Additionally, the age of onset correlates inversely with the size of the repeat [51,52,53], with a rare juvenile onset form caused by CAG repeats above 60 [54].

### 2.3. Myotonic Dystrophy Type 1

DM1 is an autosomal dominant condition caused by a CTG repeat in the 3′ UTR of DMPK, located on chromosome 19 [55]. The typical allele size of this CTG repeat is 5–34, while premutation carriers have 35–49 repeats. Although individuals with this premutation range have no documented signs of disease, their children have an increased risk of inheriting larger repeat expansions and developing symptoms [56]. The full-penetrance pathogenic alleles are those with CTG repeats of ≥50. Clinical severity of DM1 is highly variable and associated with repeat size, ranging from mild presentations with normal lifespan all the way to severe congenital forms associated with repeats into the range of thousands; this form has a strong bias toward maternal transmission [57,58]. Overall, DM1 is slowly progressive and multisystemic, with symptoms including muscle weakness, myotonia, psychiatric symptoms, MRI abnormalities, cataracts, diabetes mellitus, hypogonadism, and changes on an electrocardiogram [59]. Care is generally supportive, and like fragile X and HD, there is no cure.

*DMPK* encodes the protein myotonic dystrophy protein kinase, which is involved in maintaining skeletal muscle integrity, cardiac conduction, ion-channel regulation, and metabolism [60]. It is suggested that this expansion in the 3′ UTR of the gene causes toxic RNA accumulation, resulting in multiple molecular defects, with aberrant RNA splicing thought to be a central factor contributing to the array of disease manifestations in patients [61]. A study on the neurological impact of DM1 by Dincã and colleagues found this toxicity seems to particularly impact astrocytes by interfering with transcripts that are involved in regulating synaptic function [62].

## 3. Features and Mechanisms of STR Instability

Historically, STRs have been reported to mutate at approximately 1 × 10^−4^–1 × 10^−3^ nucleotides per generation, which is orders of magnitude higher than estimates of approximately 1 × 10^−8^ for SNVs in unique regions of DNA [63]. These mutation rates are influenced by factors such as the repeat unit size, repeat number, repeat sequence, flanking sequence, as well as sex and age [64,65,66,67,68]. For instance, longer repeat tracts are more prone to slippage during replication and, thus, expansion or contraction events [69,70,71,72]. Several recent studies have expanded our understanding of STR mutability genome-wide in human families, finding de novo mutation rates in the range of 5 × 10^−6^–5 × 10^−5^ [73]. These studies considered millions of STR loci within thousands of trios, as well as dozens of multi-generational families [19,73,74,75]. On a genome-wide scale, this translates to an estimated 62–85 de novo STR mutations per generation. We briefly discuss below some pertinent examples of important features and mechanisms of STR instability; however, we refer the interested reader to the excellent reviews that more comprehensively address this issue [2,76].

*Repeat Sequence Content*: In addition to length, sequence content has also been shown to be a determinant of instability, with important clinical consequences. In the case of *FMR1*, perfect repeats appear to be more mutable across generations. For example, the *FMR1* CGG locus contains AGG insertions; however, the loss of one or more of these AGG motifs is associated with increased intergenerational expansion [77]. In HD, loss of a CAA interruption at the end of the CAG repeat tract in intermediate allele carriers is associated with a younger age of onset as well as evidence of increased somatic instability [78]. In DM1, repeat interruptions have been associated with reduced somatic instability and milder clinical phenotypes [79,80,81]. We should note that there are some rare examples of repeat interruptions associated with increased instability and severity, potentially through increased stability of RNA hairpin structures [82,83]. Thus, sequence purity has important consequences on both germline and somatic instability, as well as clinical outcomes, but the nature of this relationship depends on the specific locus.

*Epigenetics:* The epigenetic environment may play a critical role in shaping mosaicism. For example, in fragile X syndrome, the CGG expansion is associated with not only DNA hypermethylation of the locus but also nearby histone deacetylation and histone lysine 9 methylation [84,85,86]. This heterochromatinization may have global effects, given recent work showing widespread heterochromatinization of long synaptic genes across the genome, associated with potential evidence of stepwise STR instability at distinct loci in fragile X syndrome [87]. Work in embryonic stem cells suggests switching *FMR1* off occurs early in development; using preimplantation human embryos, it was observed that undifferentiated human embryonic stem cells retain *FMR1* expression and unmethylated DNA and that *FMR1* silencing only occurs with differentiation—representing a critical developmental window during which mosaicism could arise [88]. Conversely, demethylation of the locus has been targeted in studies attempting to devise novel therapeutic strategies [89]. Artificial demethylation of the locus leads to R-loop formation (non-canonical DNA:RNA hybrids) that mediates contraction of the repeat in fragile X syndrome, which has relevance not only for understanding mosaicism, but also gene therapy approaches more broadly. In DM1, there is notable variability in methylation of the flanking sequence to the CTG repeat across individuals and tissues. This variation in methylation may moderate the instability of the repeat itself as well as biomarkers for clinical subgroups [90]. For example, Koehorst and colleagues found that unmethylated mothers passed on CTG expansions, but methylated mothers transmitted a contraction [91].

*Mechanisms of instability:* What underlies the high mutability of STRs? STR expansions are frequently linked to DNA replication, where strand slippage due to nascent and template strands misaligning during the replication process can result in changes in length [92]. However, there are also changes in length observed in post-mitotic cells; for example, there is evidence from both mouse models and human cases showing that the instability of some repeat expansions in somatic cells is influenced by the DNA repair machinery, suggesting additional distinct post-mitotic mechanisms that may contribute to disease progression. Cell-type-specific expression of repair machinery, such as mismatch repair or transcription-coupled repair, may further underlie master controls of repeat instability [93,94,95,96,97,98]. Thus, while there is evidence of critical cellular processing pathways that are likely common between STRs, there are also differences mediated by cell-type-specific gene expression profiles.

**Figure 2:**
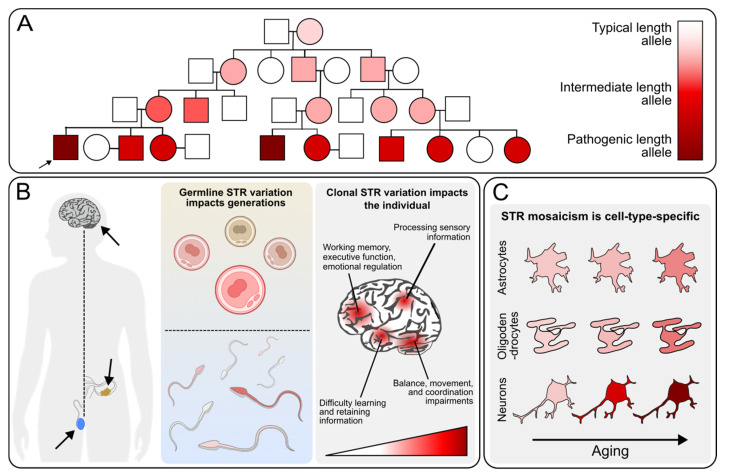
Illustration of the impact of STR instability over generations and the clinical significance of STR mosaicism that can be found within individuals. (**A**) Example pedigree demonstrating expansion across generations, particularly in the female germline for fragile X. (**B**) Mosaicism in the brain can impact clinical phenotypes of neurological disorders, while germline variation impacts generational changes, which can lead to differential expansion in sperm (HD) and egg cells (fragile X, DM1) depending on the specific STR. The right panel demonstrates the function of different brain regions, which could theoretically be impacted by clonal variation. (**C**) Somatic mosaicism, in some cases such as Huntington disease, may be developmentally dependent and cell-type-specific but not necessarily tied to proliferative status.

STR instability can result in changes in disease severity across generations due to mosaicism in germ cells (Figure 2A), i.e., anticipation. Likewise, mosaicism within an individual can also lead to variability across cells within an individual beyond the germline, theoretically impacting disease severity depending on where in the body this occurs (Figure 2B). Finally, given cell-type-specific expression of factors, such as DNA repair machinery, this variability may differ across different cell types, which could lead to specific, predictable phenotypes (Figure 2C).

## 4. Mosaicism and STRs

In the past two decades, sequencing improvements have allowed us to appreciate the mosaic nature of the human body and how this affects health and disease (See Section 6 below). Broadly speaking, mosaicism (of different forms of genetic variation and not just STRs) has been shown to increase with age and contribute to cardiovascular disease, liver disease, changes in inflammation status, schizophrenia, and cancer [99,100,101,102]. Mosaic chromosomal alterations, such as aneuploidy and large-scale copy number variations (CNVs), have been associated with neurodevelopmental and neurodegenerative disorders, including autism, Alzheimer’s disease, and Parkinson’s disease [103,104,105,106,107]. Additionally, the accumulation of somatic mutations in stem cell populations has been proposed as a mechanism underlying age-related decline and regenerative capacity [108].

STR mosaicism has been more challenging to study due to obstacles in accurately measuring these repetitive regions. In the context of fragile X, although both length and methylation mosaicism are frequently described, we know surprisingly little about how mosaicism contributes to clinical phenotypes (see below). There have been several case reports identifying mosaicism of the CGG repeat length, where some cells carry the full mutation allele, and others carry premutation or normal range alleles [109,110,111]. There is also methylation mosaicism, where cell populations may contain either methylated, unmethylated, or partially methylated *FMR1*. There is evidence that size and methylation mosaicism alter FMRP production, presumably driving clinical phenotypes [112,113]. However, given the differing phenotypes of premutation and full mutation carriers, as well as the difficulty of studying the human brain directly, the full clinical implications of *FMR1* mosaicism remain to be seen. It is tempting to speculate that the frequency and complexity of mosaicism in fragile X-related conditions could drive the impressive clinical heterogeneity observed in these disorders, even in carriers with measured expansions of similar sizes.

*FMR1* is located on the X-chromosome and is therefore subject to the stochastic process of X-chromosome inactivation [38,39]; this process maintains gene dosage in XX female cells by turning off one X-chromosome early in development, and female carriers—as a consequence—are naturally mosaic for which X chromosome is active. The activation ratio, which is the percentage of cells that carry the benign *FMR1* allele as active, likely contributes to variable disease severity in females with fragile X-related conditions. In fact, there is evidence that the activation ratio correlates both with cellular and clinical phenotypes [114]. However, most studies that assess the activation ratio in female carriers primarily study blood, which may differ from patterns within the human brain. Thus, studies of peripheral tissues may underestimate the true impact of activation ratio on clinical outcomes. Understanding *FMR1* dynamics in relation to sex is even more important when one considers an increased predilection for instability within the female germline [115].

## 5. Clinical Impact of STR Mosaicism and Disease Severity

The following studies shed light on the intricate interplay between CGG repeat size and methylation patterns in shaping the clinical spectrum of FXS phenotypes, as well as the opportunities for future progress.

Fernandez and colleagues presented two unrelated families, each having one child with FXS and one sibling with an FXS-like phenotype [116]. Each of the siblings presenting with the FXS-like phenotype was revealed to have mosaicism in CGG repeat size, methylation across different tissues, or both. In Case 1, the identified alleles ranged from premutation to full mutation sizes, with varying levels of methylation. In Case 2, only premutation sizes were identified, with unexpected evidence of some methylation. The authors proposed that the size diversity of the repeat alleles and their methylation status have direct effects on mRNA and protein levels, which could explain the FXS-like phenotype [116].

Larger cohorts have also provided convincing evidence of the importance of mosaicism in fragile X-related conditions. Meng and colleagues conducted a comprehensive analysis of 487 males with FXS, illuminating the differential impact of methylation and size mosaicism on cognitive and behavioral outcomes [117]. Overall, they found evidence of size and/or methylation mosaicism in over 30% of the cohort. Their findings showed a milder phenotype in individuals with methylation mosaicism compared to those without, while size-related mosaicism showed less pronounced associations. Limitations of this study, and many others studying the clinical relationship of mosaicism, include that mosaic status was inferred from blood testing, which may or may not correlate with brain-specific changes [117]. Pretto and colleagues examined 18 individuals with FXS, 13 of whom were identified to have mosaicism when comparing peripheral blood mononuclear cells (PBMCs) and fibroblasts [110]. Among the mosaic cases, the study identified variation in methylation patterns and CGG repeat sizes between the tissues, with higher levels of *FMR1* mRNA being found in the blood than in fibroblasts. Notably, they found that cognitive improvement scores were positively correlated with the subtle increases in FMRP levels in the mosaic cases, highlighting the significance of lower methylation compared with full methylation, full mutation alleles, on clinical outcomes [110]. Interestingly, a study by Baker and colleagues found that although premutation mosaicism was associated with improved intellectual functioning in individuals with fragile X syndrome, autistic features were similar regardless of the presence of mosaicism, once controlling for IQ [118]. Mosaicism is also well described in the *FMR1* premutation. In a recent study of premutation females, mosaicism was identified in the buccal tissue in the majority of the participants and associated with unexpected health outcomes [119]. Thus, size and methylation mosaicism in fragile X-related conditions may be the rule, as opposed to the exception, and they impact some domains of clinical severity.

In HD, instability of CAG repeats in both the testes and brain has been described for over 30 years [120]. Larger repeat lengths are associated with earlier disease onset, making somatic instability a marker for disease pathogenesis [121]. Larger tracts are also observed to become more unstable in postmitotic neurons [122]. Somatic instability is thought to be caused by differences in DNA repair proteins and pathways, for instance, mismatch repair due to the formation of secondary structures [123]. HD is also known to exhibit meiotic instability, as it is observed to contribute to repeat size growth over generations, particularly in the male germline [124,125].

In HD, somatic expansion has been shown to determine clinical outcomes beyond the germline expansion size itself [126]. Recent work [127,128] has also made tremendous advances in providing support for the Kaplan hypothesis [129], which states that the expansion of TRDs beyond pathogenic thresholds in specific cell types mediates clinical progression. Specifically, Handsaker and colleagues [128] used single-cell genomic approaches in human brain tissue and found that it was not the progression beyond 40 repeat units that led to aberrant gene expression, but rather, the cell-specific somatic expansion of these alleles to greater than 150 repeat units led to widespread gene dysregulation and markers of senescence and apoptosis in striatal neurons. Their work suggests that HD reflects a lifelong process of expansion in vulnerable cell types and has profound implications for revolutionizing our understanding of diagnosis and therapeutics more broadly for TRDs [130]. Other neuronal subtypes in HD have also been demonstrated to have unique vulnerability to pathogenic somatic expansions, including layer 5a pyramidal neurons [131]. However, these authors found that other, more resilient neuronal subtypes also demonstrated evidence of STR expansion and posited that altered connectivity is an additional “hit” necessary for neuronal loss. Why some neurons are uniquely vulnerable to expansion is an important critical area of open investigation.

In DM1, there is well-established instability of the CTG expansion in both somatic tissues and germline. For example, the sperm from male individuals with DM1 show extensive expansion size variability across individuals [132]. Additional analyses taking advantage of data from preimplantation genetic diagnostics found that oocytes and embryos from females with DM1 displayed larger increases in repeat numbers, while the sperm and embryos from males with DM1 had smaller increases at ~50–100 repeats [133], consistent with primary transmission in the female germline.

A longitudinal study observed how CTG repeat mosaicism changed over time in the blood of 43 DM1 patients [134]. They found that the range of repeat sizes and modal allele length generally increased over time, with the rate of change affected by multiple factors that included inherited progenitor allele length, age at sampling, time interval between samples, and sex of the patient. Importantly, individual variation in rates of expansion was associated with differences in age of disease onset. Several studies analyzing the somatic tissue-specificity of CTG expansion rates have shown consistent results of repeat lengths being much larger in muscle than what is observed in blood [135,136,137], highlighting the significance of understanding mosaic patterns when considering patient prognosis.

Collectively, these studies underscore the significance and frequency of mosaicism in shaping the clinical heterogeneity in fragile X, HD, and DM1. The findings emphasize the importance of testing multiple tissues and highlight potential challenges in genetic counseling and management for such cases. For example, could cryptic mosaicism influence the success of clinical interventions, thus necessitating more comprehensive characterization in clinical trials? These case examples also serve as a foundation for the application of similar studies in other TRDs in which mosaicism may be contributing to clinical phenotypes. This is critical as somatic mosaicism is a common feature of numerous, albeit less well-characterized REDs [138].

## 6. Current STR Detection Methods and Limitations

Several approaches are used to detect STRs (Table 1); however, their reliability, cost, and resolution of mosaicism detection vary. In rare disease genetics, gene panels or short-read whole-exome and genome sequencing remain the standard of care. However, pathogenic STR expansions are challenging to accurately size in short read datasets when the expansion exceeds the insert read length. This necessitates separate, targeted assessments for TRDs depending on clinical suspicion. Established current clinical methods include repeat-primed polymerase chain reaction (PCR) and Southern blot, but these are targeted and cannot accurately determine cell fraction in cases of mosaicism. Triplet-repeat primed PCR is perhaps the most used given the relative ease of implementation; although the specifics may vary, in general, it uses a third fluorescently labeled, repeat-specific primer that generates a characteristic trace following capillary electrophoresis. Multiple alleles can be clearly resolved, including very large alleles and mosaic alleles. Adaptations of this method include the incorporation of a methylation-sensitive enzyme, which allows for inference of methylation state. However, there remains a bias toward the amplification of smaller alleles, which means one cannot draw conclusions about the relative proportion of mosaic alleles. Specifically, very long alleles may demonstrate only small fluorescent peaks and yet comprise a substantial mosaic cell fraction. Furthermore, because of this bias, large expansions representing mosaic cell fractions less than 5–10% may not be reliably detected [139]. Furthermore, there is no base pair resolution, which, as described above, can have important effects on instability [140]. However, PCR “stutter”, visualized as dips in the electropherogram, can be used to indirectly infer the presence of repeat interruptions.

In the past decade, several key advances in STR detection have created a paradigm shift in the field. First, several computational methods have been developed capable of genotyping germline STRs in short-read data, both targeted and genome-wide. These pipelines have unearthed new horizons in the detection of short tandem repeat disorders from short-read sequencing data; however, they remain limited in their sensitivity for some loci and struggle to accurately resolve very long expansions [1,141,142,143,144,145,146]. Nonetheless, there have been encouraging findings regarding both the specificity and sensitivity of such tools in identifying pathogenic repeat expansions compared to gold-standard approaches [147]. Mosaic STR variation is even more difficult to call from short-read data, although there have been recent advances in this domain as well, including prancSTR [148]. However, prancSTR is most helpful for single high-frequency mosaic alleles, which may not represent true patterns of mosaic variation for large clinically relevant expansions. Overall, these tools open up new opportunities for STR interrogation on countless pre-existing large-scale datasets, particularly for research applications in which accurate sizing may be less critical.

The second major advance that has altered our ability to detect STR expansions is long-read sequencing. Single-molecule real-time (SMRT, PacBio) sequencing and nanopore sequencing [140] read through thousands of nucleotides with sufficient accuracy, even within repetitive regions. Pacbio produces shorter but more accurate reads up to approximately 20 kilobases, while nanopore sequencing can achieve ultralong reads potentially spanning millions of bases. Thus, both are well suited to detect even the longest repeat expansions. The emergence of long-read sequencing and genotyping methods, such as TRGT, LongTR, and Straglr, has allowed for STR detection and visualization from these datasets [149,150,151]. Another advantage of these long-read sequencing approaches is the direct sequencing of native DNA. For example, SMRT sequencing infers 5-methylcytosine modifications through the detection of polymerase kinetics that are dependent on the presence of base modifications. Likewise, nanopore sequencing can be trained to detect various modifications from the raw conductivity data. There have been recent studies demonstrating the use of long-read sequencing to accurately genotype large STR expansions in a high-throughput capacity [152]. However, standard, genome-wide coverage may be insufficient to detect mosaicism of large expansions. Thus, methods to first enrich the targeted regions, such as with amplification-free Cas9-mediated enrichment and nanopore’s adaptive sampling to perform real-time target selection [153,154,155,156], allow for higher sequencing depth of repeat expansions, which enables mosaicism detection at a reasonable cost. Other methods, such as indirect sequence capture, are also being proposed to allow for high coverage assessment of repeat expansions [157]. These approaches as a whole, which allow for high coverage sequencing through even the largest repeats, are poised to accelerate our understanding of STR mosaicism and are likely to become standard in the field.

## 7. Future Directions on the Clinical Impact of STR Mosaicism

The above cases illustrate the importance of considering STR mosaicism in both cellular and clinical phenotypes, although important gaps in our understanding remain. While studies have reported the presence of STR mosaicism in various disorders, the overall prevalence and patterns of mosaicism across different STRs and tissues are not well-established [158,159]. Even in fragile X-related conditions, we know little about how mosaicism varies by cell type and brain region in the human condition as compared to HD. More comprehensive studies are needed to determine the frequency and characteristics of STR mosaicism in both health and disease, as well as across different cells and tissues in the human condition. STR mosaicism can complicate diagnostic testing and interpretation, as the detection of mosaicism may depend on the accessibility of the tissue sampled and the sensitivity of the testing method [160]. This is of particular relevance to neurologic disorders, as brain tissue is typically not available for study in a clinical population. Rather, information regarding mosaicism may be inferred from the sampling of peripheral tissues, such as blood or skin. A better understanding of the features and patterns of mosaicism from even the most fundamental perspectives should prove helpful, for example, in determining if certain peripheral tissues might better mimic patterns in the brain. Additionally, most studies on STR mosaicism have focused on coding regions, while the prevalence and consequences of STR mosaicism in non-coding regions of the genome are less explored. Investigating STR mosaicism in regulatory regions and its potential effects on gene expression and disease pathogenesis is an important area for future research. Unlike single-nucleotide variants, in which mosaicism can be represented as a binary outcome in a particular cell, STR mosaicism is potentially drastically more complex, with a large range of STR lengths potentially co-existing in a single individual. This nuance needs to be considered in both basic science studies and clinical statistical modeling, as groups are already attempting to do [127]. Finally, and most importantly, we have provided an in-depth overview of three disorders as case examples of the scope of the importance of mosaicism in STRs. However, there are dozens of disorders caused by STR expansions, and in many cases, mosaicism is also important in those contexts. Given that there are likely at least some shared mechanisms driving STR instability, research into these individually rare disorders will likely result in an improved understanding of STR biology more broadly (see Section 3 above).

Although the American College of Medical Genetics incorporates consideration of mosaicism in genetic testing for TRDs such as fragile X [160], our ability to use mosaicism to counsel a patient remains more limited, e.g., what does mosaicism mean for the individual? Establishing evidence-based guidelines for genetic counseling of individuals and families with STR mosaicism will require improved knowledge about the natural history and the mechanisms and consequences of mosaicism in these disorders across the lifespan and across tissues. Given the technical challenges in detecting low-level mosaicism and the complex inheritance patterns of repeat expansion disorders, this is not a trivial task [32,161]. For example, in FXS, the presence of methylation mosaicism can complicate the interpretation of genetic test results and the assessment of disease risk [32]. Moreover, the genetic counseling of individuals and families with STR mosaicism requires a nuanced understanding of the clinical and psychosocial implications of variable repeat expansion [32,161]. The unexpected finding that premutation females can be mosaic for the full mutation [119] is a prime example of the importance of empirically studying the effects of mosaicism on clinical outcomes in large-scale and longitudinal clinical cohorts.

Improvement of STR sequencing will clarify the range of allele sizes, resulting in variable expression patterns that cause disease [1,119,162]. Comprehensive testing for STR mosaicism is crucial for informed reproductive decision-making, as it can have significant implications for family planning and prenatal testing. A better understanding of the precise mechanisms underlying mosaicism opens opportunities for the development of tandem-repeat targeted CRISPR gene editing, personalized antisense oligonucleotide, biologics, and other small molecule therapeutics [12,163]. Furthermore, given efforts to develop gene therapy to reintroduce *FMR1* in a subset of cells—which essentially creates a unique situation of mosaicism for *FMR1* expression dissociated from the repeat expansion itself—understanding the nuances of the effects of mosaicism could take on even new, unexpected relevance.

## 8. Conclusions

This review encompasses the role of STR mosaicism in human disease with a focus on fragile X, HD, and DM1 and highlights the importance of discerning mosaicism in determining disease severity and progression and developing potential therapeutics. Appropriate STR resolution methods are critical in identifying the repeats themselves and their mosaic patterns. As sequencing methods and technologies continue to advance, allowing for more efficient and cost-effective analyses, our ability to detect STR mosaicism will improve, with the current emergence of long-read sequencing set to become the standard tool in comprehensive STR analysis [148].

Further exploration into identifying the mosaic patterns of short tandem repeats has important implications for refining disease prognosis and customizing treatment interventions. Currently, the influence of STR mosaicism on disease progression and response to potential therapies is largely in its infancy and is an area that demands longitudinal studies that track changes in STR mosaicism over time and across tissues as a means to assess the impact on clinical outcomes and treatment response.

## Figures and Tables

**Table 1:** Overview of techniques to identify STRs and mosaicism. * Although triplet-primed PCR can detect multiple alleles, preferential amplification of smaller peaks limits mosaicism quantification and sizing of large alleles. ** Southern blotting can detect mosaicism if cell fraction is high enough but, again, may be limited with larger expansions in a smaller number of cells. *** New algorithms have been deployed to detect mosaicism of STRs in short-read data; however, this remains an emerging area. **** Caution must be exercised in interpreting mosaicism in whole genome sequencing, even with long reads. Standard 25× whole genome coverage may be insufficient to detect the presence of mosaicism for large expansions.

Method	Triplet-Primed PCR	Southern Blotting	Short-Read Sequencing	Long-Read Sequencing
Description	Modified polymerase chain reaction (PCR) using multiple primers, including one targeting repeat. Produces different-sized DNA due to multiple annealing sites within the repeat region. Visualized by capillary electrophoresis. Digestion with a methylation-sensitive enzyme can be incorporated for methylation detection.	DNA digestion, gel electrophoresis DNA detection	DNA fragmentation followed by massively parallel sequencing. Bioinformatic processing uses alignment algorithms to resolve the repetitive STR and flanking region.	High molecular weight DNA is used; single-molecule real-time or nanopore sequencing provides highly accurate, kb long reads with base pair and methylation level resolution. Bioinformatic processing uses specialized alignment algorithms to resolve the repetitive STR and flanking region, as well as DNA methylation status.
Cost	$	$	$$	$$$
Accuracy	Lower for larger STRs	Lower for larger STRs	Lower for larger STRs	High
Limitations	No sequence information, and smaller amplicons disproportionately amplified	No sequence information, labor intensive, and less scalable than other methods	Less reliable for large or complex STR expansions	Less widely available and most expensive
Advantages	Commercial kits available	No amplification requirement	A large number of datasets amenable to analysis	Accurate and precise. Combination with other approaches (amplification-free enrichment) permits high-coverage analysis.
Methylation Detection	✔	✔	✔	✔
Mosaicism Resolution	✔ *	✔ **	✔ ***	✔ ****

## Data Availability

No new data were created or analyzed in this study.
